# Editorial: Real world evidence, outcome research and healthcare management improvement through real world data (RWD)

**DOI:** 10.3389/fpubh.2022.1064580

**Published:** 2023-01-04

**Authors:** Carolina Varela-Rodríguez, Nicolás Rosillo-Ramirez, Gabriel Rubio-Valladolid, Pedro Ruiz-López

**Affiliations:** ^1^Quality of Care Unit, Hospital Universitario 12 de Octubre, Madrid, Spain; ^2^Appropriatness of the Clinica Practice and Outcome Research, Instituto de investigación biomédica del Hospital Universitario 12 de Octubre I+12, Madrid, Spain; ^3^Preventive Medicine Department, Hospital Universitario 12 de Octubre, Madrid, Spain; ^4^Mental Health Department, Hospital Universitario 12 de Octubre, Madrid, Spain

**Keywords:** real-world data, real-world evidence, electronic health records, PROM, PREM, CROM, medical epistemology

The systematic use of Real World Data (RWD) in clinical practice and the real-time application of clinical epidemiology to clinical healthcare and management decision-making processes represent one of the healthcare innovations with the most significant possible impact on the sustainability of healthcare systems and the appropriateness of clinical decisions (1–4). For this to be possible, the quality of the data, the analysis performed, and their availability must be adequate to answer the science question; it should be systematically recorded and compliant with the FAIR principles: findable, accessible, interoperable and reusable.

Nowadays, Electronic Health Records (EHR) potentially allow the recording and availability of truthful, accurate, and timely data for appropriate and reliable informed decision-making. Clinician-reported patients' health data, Medical Devices-reported data, and Administrative Databases nourish those medical records; even patients' quality of life (QoL) information is being increasingly recorded (Figures 1B,C). However, this opportunity hinges on the quality of the data; if the data has quality and reliability, the conclusions obtained provide value to the decision-making process; if this is not the case, it can generate biases that jeopardize the appropriateness of the decisions.

**Figure 1:**
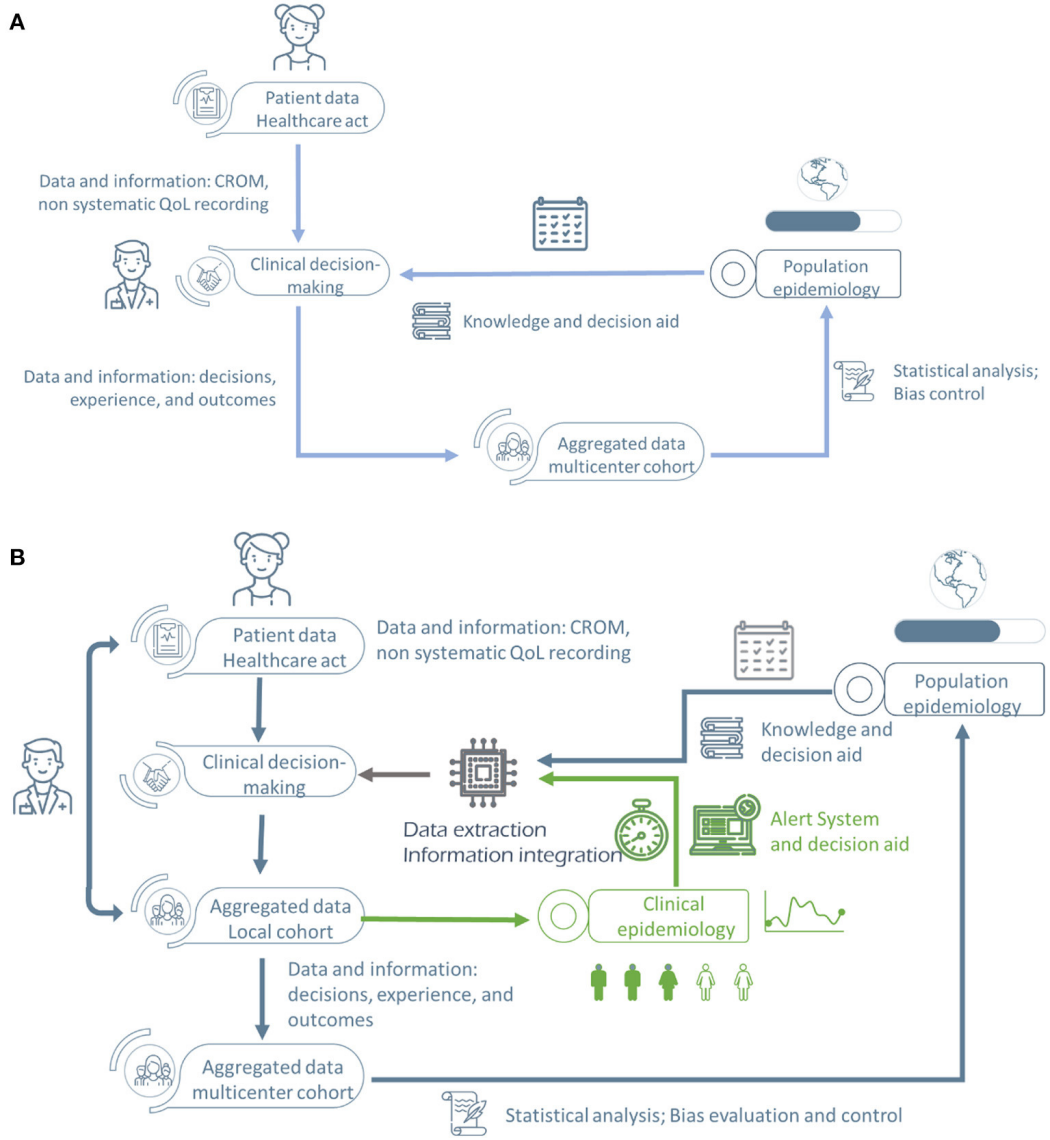
Epistemological change and redesigned Healthcare Information Systems (HIS) within the requisites of the RWE paradigm. **(A)** Traditional medical epistemological paradigm. **(B)** New medical epistemological paradigm with “real-time” incorporation of the scientific evidence produced during the healthcare act [QoL, Quality of Life; RWE, Real World Evidence; CROM, clinician-reported outcome measures (clinical indicators)].

The new epistemological paradigm within learning organizations implies having procedures and methods to evolve from data acquisition to evidence generation. For that, standardization, normalization, and structured data recording are prerequisites to an appropriate analysis and comparison of the data and, therefore, to transform data into knowledge. For that, there are four critical aspects of evidence-generating information systems (Figure 2): clinical indicators (CROM and MDRM), quality of life measures (PROM), perceived quality and experience indicators (PREM), social determinants of health information (ADB and PROM) from primary sources. Up to date, the relationship between citizens with healthcare information systems (HIS) does neither include patient information directly in the HIS nor is patient experience systematically recorded. As shown in Figure 1A, health professionals introduce patient health outcomes and experience information, including biases and missing information.

**Figure 2:**
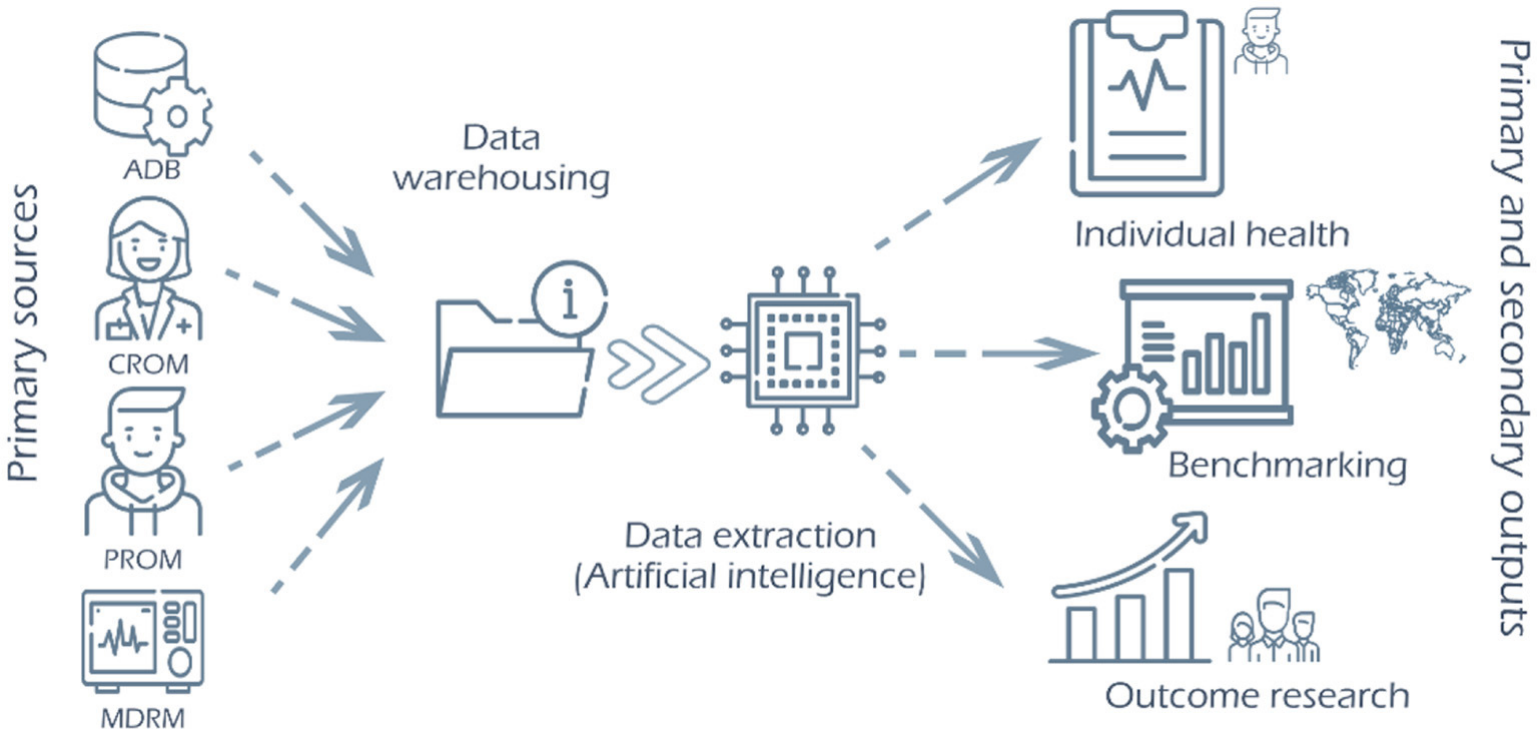
New Health Information System structure implies the inclusion of data from primary sources (professional, patient, administrative databases and medical devices, including wearables) in centralized data warehousing and the development of means to extract the different information outputs from a unique data entry, answering to individual healthcare needs as much as the information requirements for administrative, public health and research purposes [QoL, Quality of Life; RWE, Real World Evidence; CROM, clinician-reported outcome measures (clinical indicators); PROM, patient-reported outcome measures (quality of life health outcome indicators); PREM, patient-reported outcome measures (patient experience indicators); MDRM, Medical Devices-Reported Measures (clinical indicator measured by medical devices); ADB, Administrative Databases (patient information from administrative databases include social determinants of health)]. Source: Modified with author's permission from Varela-Rodríguez et al. (5).

This paradigm change would impact the whole healthcare/society relationship and medical epistemology. The traditional XX-century Epistemological Paradigm in Medicine (Figure 1A) introduces data slowly in the system, generating knowledge as slowly as the data is gathered. However, this knowledge management system shows more fragmentation. Still, it has several ways to check the scientific evidence underneath the results and conclusions, ensuring comparability and reliability, such as peer review and scientific forum discussion. Keeping biases and errors under control was very time-consuming.

The New Epistemological Paradigm in Medicine, represented in Figure 1B, changes those facts, incorporating evidence from clinical studies into the decision-making process in real-time. Real-time is understood to be within reasonable parameters in the care process (days or weeks). This epistemological change, illustrated in Figure 1B, is one of the main challenges to be faced by the healthcare systems. Information systems are critical elements in this evolution, together with the adequate training of professionals and citizens (present and future patients, after all). With this Research Topic, we wanted to capture at least part of the complexity of this transformation.

The EHR enables quality data collection and should be the source of information for primary (healthcare, quality and safety, management) and secondary use of data (clinical research) (Figure 2). Therefore, the following should be warranted, normalization and standardization of data and codification with international standards (ICD-10, SNOMED, LOINC...) so the portability of data and data sharing becomes a reality. Adequate data recording, analysis and visualization tools should be developed at different levels of decision-making (clinical management, quality and safety management, and health management). It allows for response to the various information needs or “outputs” (Figure 2) of the institution and its professionals and the creation of automated alerts (triggers) to detect risks and security incidents. Furthermore, all this has to be done at any geographical location or sociodemographic situation within the current global health reality.

Thus, Saha et al. brought us the paramount need to teach and educate professionals (intermediate and final users of the HIS), giving them the knowledge and tools to adapt to these abrupt changes appropriately. Never in history change have been so fast, and healthcare professionals are supposed to adapt as quickly and adequately to them as possible, often without completely understanding the true nature or consequences of the innovations they use. On the other hand, we will need technological innovations to support those changes; Marks et al. brought us the software to make data recording possible in no-so-easy circumstances and with significant public health implications. Moreover, this quick data recording and analysis has to help with the rapid changes in healthcare logistics. If we want to be effective and do the appropriate procedure or treatment for the right patient at the right moment, we must have the resources to do so. Solís-Díez et al. showed us how this new technological paradigm could help. Furthermore, Du et al. exemplify how including patients and citizens in healthcare institutions aids continuous improvement, transformation, and resource allocation optimization.

Last but not least, Buja et al. and He et al. showed us how, when data is appropriately recorded and aggregated together, when methods are carefully applied to analyze those data, conclusions can be drawn to help the decision process with our present and future patients.

Finally, our topic raised another challenge: the inequity in health and how innovations and technology could help to increase or reduce this injustice. Saha et al. showed how interventions in middle-income countries could be on top of innovations and impact population health. Marks et al. showed how technical obstacles could be saved; for example, even in zones with no access to the internet, appropriate data collection can be done and then warehoused asynchronously.

The manuscript introduces the articles of the Research Topic, contextualizing the current situation of health information systems within a new paradigm of medical epistemology and data gathering. This new paradigm includes:

- Inclusion of data from the primary sources.
- Inclusion of patient perspective, including outcomes and experience.
- Inclusion of social determinants information.
- Education of patients and professionals within the new paradigm.
- Empower and activate all stakeholders in the data gathering.
- Bias and errors control.

The editorial proposes a restructured Health Information System to respond to the new paradigm in medical epistemology.

## Author contributions

CV-R has contributed to the original draft preparation's conceptualization and writing and editing. PR-L and GR-V have contributed to the manuscript's conceptualization, critical reading, and correction. NR-R has contributed to the essential reading and review of the manuscript. All authors have contributed to reviewing. All authors contributed to the article and approved the submitted version.
