# Supplementary material for: Editorial: Real world evidence, outcome research and healthcare management improvement through real world data (RWD)
Source: Front Public Health. 2023 Jan 4;10:1064580. doi: 10.3389/fpubh.2022.1064580 (PMC9846327; doi:10.3389/fpubh.2022.1064580)
Supplement: Supplementary file 1 [file Data_Sheet_1.docx]

**SUPPLEMENTARY MATERIAL:**

Table S1: Update of the projects developed in the Hospital Universitario 12 de Octubre:

| **Project** | **Insights** | **Key variables recorded (Minimum)** | **Team composition** | **Cohort * (N)** | **Follow-up time**  **(current phase)** |
| --- | --- | --- | --- | --- | --- |
| Breast Cancer | Development and design of structured forms for CROM  Development and design of structures forms fos PROM  Development and design of PREM  Validation of questionnaires PREM  Team organisation methods  External communication  Internal communication | 203 | Three clinical leaders  15 members of the unit  One managerial leader  Two economic analysis professionals  Data manager and Data Scientist  Coordinator  Seven patients | YES (165) | 12 months  Evaluation and adoption |
| Lung Cancer | Development and design of structured forms for CROM.  Development and design of structures forms fos PROM  Development and design of PREM  Validation of questionnaires PREM  Team organisation methods  External communication  Internal communication | 143 | Four clinical leaders  15 members of the clinical unit  One managerial leader  Two economic analysis professionals  Data manager and Data Scientist  Coordinator  Five patients and three carers | YES (108) | 12 months  Evaluation and adoption |
| ARMD | Lean analysis of the process  Community creation and communication.  Accessibility to PROM and PREM questionnaires (low visual acuity) | 48 | Two clinical leaders  Ten members of the service  One managerial leader  Two economic analysis professionals  Coordinator | YES (0) | NA  Piloting |
| IBD | Community communication  Interoperation with national registries. | 92 | Four clinical leaders  15 members of the unit  One managerial leader  Two economic analysis professionals  Data manager  Data Scientist  Coordinator | NO | NA  Data and tool adaptation |
| COVID-19 | ICHOM Standard set definition - Delphi study | NA | Two specialists  Coordinator | YES (>800) *** | NA |

*At the date of writing **Cohort = we have means to identify the patients with inclusion criteria. However, due to some technical problems, in the ARDM, we have not yet recruited the patients. *** Partially implemented.

Table S2: Software used in the implementation

| **Software** | **Version** | **Description** | **Task(s) related** | **Licenses** |
| --- | --- | --- | --- | --- |
| Microsoft Windows | Windows 10 Pro  1909 | Operative system | Operative system | Institutional license |
| LINUX | Ubuntu LTS | Operative System | Open source operative system | OpenSource |
| Microsoft Office* | Microsoft Standard 2016 | Offimatic package | Process analysis  Documentation of the project | Institutional license |
| OpenOffice* | 4.1.11 | Offimatic package | Process analysis  Documentation of the project | OpenSource |
| PowerBI | 2.99.782.0 64 bit | Dashboard design and creation | Dashboard design and creation | Free version for Dashboard design (freemium) |
| Addsum (HOPES) | 5.0 | A platform for data collection and visualisation | PROM design  PROM collection | Development license |
| Panel.Health | Beta test | Experience data collection platform.  Iterative PREM design. | PREM design  PREM collection | Development license |
| Redcap | 10.6 | Interface for data inclusion in a protected database. Different users can fill it | Data recording and warehouse | OpenSource |
| Google Drive* | NA | Project documentation and cooperative modification of the documents | Document sharing and warehousing | Free |
| Dropbox* | NA | Project documentation and cooperative modification of the documents | Document sharing and warehousing | Free version |
| Sharepoint* | NA | Project documentation and cooperative modification of the documents | Document sharing and warehousing | Institutional License |
| Miro | NA | Collaborative canvas | Process analysis | Free basic version (freemium) |
| Trello* | NA | Digital Kanban tool for team communication and organisation | Team communication and tasks allocation | Free basic version (freemium) |
| Slack* | NA | Digital Kanban tool for team communication and organisation | Team communication and tasks allocation | Free basic version (freemium) |
| Slidesgo | NA | Creative commons Slide templates repository | Project documentation  External and internal communication | Free access |
| Piktochart* | NA | Infographic design | Project documentation  External and internal communication | Free basic version (freemium) |
| Canva* | NA | Infographic design | Project documentation  External and internal communication | Free basic version (freemium) |
| Pixabay | NA | Creative commons Slide templates repository | Project documentation  External and internal communication | Free basic version (freemium) |
| Powtoon | NA | Video Design | Project documentation  External and internal communication | Free basic version (freemium) |

Table S3: Implementation process indicators help to evaluate the implementation progression success.

| **Implementation process indicators** | | | |
| --- | --- | --- | --- |
| **Implementation aspect** | **Name** | **Description** | **Formula** |
| Implementation process quality | Team diversity | Evaluation of the interdisciplinary of the science team | Number of different knowledge areas included |
|  | Professional engagement | Project meetings participation | Number of persons participating in the meeting x 100/persons invited to participate |
|  | Patient engagement | How many of the patients use their time to answer a voluntary and anonymous questionnaire to improve the system | Number of patients that fill up at least one PREM. The number of patients that fulfill at least one PREM x 100/Total patient on the Cohort. |
|  | Performance of the continuous improvement cycle | How many of the team's improvements detected and agreed upon in the meetings are implemented. | Number of agreed compromises agreed in teams meetings x 100/total of improvements developed |
| Data recording quality | Success percentage | Patients included in the Cohort fulfilling inclusion criteria | Number of patients included in the cohort x 100/ total patients with inclusion criteria. |
|  | First intention data fulfillment | Fulfillment of CROM without help from the data manager. Variables fulfill per patient EHR/Total variables from the dataset (CROM). | Number of data recorded without data manager intervention x 100/ all the data that should have been recorded |
|  | Degree of data recording automation | Variables automated in the system for extraction. | Number of variables fulfill automatically per patient HER x 100/Total variables from the dataset (CROM) |
|  | Availability of the cost information | Costs per patient included in the evaluation of value. | Costs available per patient/total costs considered in the cost dataset. |
| Data quality and patient follow-up | PROM Recurrence ratio | Patients lost for the Cohort over time | Number of patients that answer the previous PROM x 100/patients that answer the following PROM. |
|  | Follow-up calls | Data completeness without the intervention of a data manager | Number of follow-up calls made by the data manager both to patients and professionals. |
| Feedback and improvement | PROM use | Professionals that consult PROM results before the patient visit. | Number of professionals access to the PROM x 100/total of professionals caring for the patients. |
|  | Areas of improvement detection | Number of times that the systematic analysis of data triggers an alert for patient care or process improvement | Number of alerts identified |
|  | Alerts response | Alerts analysed and answered by the professional team. (e.g., schedule a new appointment with mental health once depression or anxiety levels rise in PROM) | Number of responses to an alert (e.g., schedule a new appointment with mental health once depression or anxiety levels rise in PROM) x 100/Total number of alerts |
|  | Continuous improvement opportunities | Improvement actions put in place identified using the PROM and PREM responses. | Number of improvements derived from PREM and PROM analysis. |
| Managerial implication | Professional substitution | When a key role is out of the project due to holidays, sick leaves, or any other circumstance, he or she should be substituted | Days without a professional role (e.g., case manager) x 100/total of the professional leave days |
